# Functional Analysis of *CbbHLH35* Reveals Its Role in Drought and Cold Stress Tolerance in *Caladium bicolor*

**DOI:** 10.3390/plants15071120

**Published:** 2026-04-06

**Authors:** Yinzhu Cao, Yan Huang, Huafeng Wu, Ximeng Yang, Fan Li, Shenchong Li, Shunzhao Sui

**Affiliations:** 1 Key Laboratory of Agricultural Biosafety and Green Production of Upper Yangtze River (Ministry of Education), Chongqing Engineering Research Center for Floriculture, College of Horticulture and Landscape Architecture, Southwest University, Chongqing 400715, China; yinzhu202108@163.com (Y.C.); 18725652900@163.com (Y.H.); swuwhf@126.com (H.W.); 2 Chongqing Key Laboratory of Forest Ecological Restoration and Utilization in the Three Gorges Reservoir Area, Chongqing Academy of Forestry, Chongqing 400036, China; xm2020@email.swu.edu.cn; 3 Key Laboratory for Flower Breeding of Yunnan Province, National Engineering Research Center for Ornamental Horticulture, Floriculture Research Institute, Yunnan Academy of Agricultural Sciences, Kunming 650205, China; lifan@yaas.org.cn

**Keywords:** *Caladium bicolor*, bHLH transcription factor, *CbbHLH35*, drought stress, cold stress

## Abstract

*Caladium bicolor* is an important ornamental foliage plant; however, its tropical origin makes it highly sensitive to environmental stresses such as drought and low temperature, which limits its cultivation and industrial development. Basic helix–loop–helix (bHLH) transcription factors play key roles in plant responses to abiotic stresses, but their functions in *C. bicolor* remain largely unknown. Here, a bHLH transcription factor gene, *CbbHLH35*, was cloned from *C. bicolor*, and its sequence characteristics, subcellular localization, expression patterns, and potential roles in stress responses were analyzed. The results showed that CbbHLH35 contains a conserved bHLH domain and is localized in the nucleus. qRT-PCR analysis revealed that *CbbHLH35* is expressed in different tissues, with the highest expression in tubers, and is significantly induced by methyl jasmonate (MeJA), abscisic acid (ABA), drought, and low-temperature treatments. Transgenic *C. bicolor* plants overexpressing *CbbHLH35* were generated and subjected to drought and cold stress. Compared with control plants, the overexpression lines showed higher chlorophyll content and POD activity but lower electrolyte leakage and MDA content, indicating enhanced drought and cold tolerance. These results suggest that *CbbHLH35* plays a positive role in regulating drought and cold tolerance in *C. bicolor* and represents a promising candidate gene for the molecular breeding of stress-resistant cultivars.

## 1. Introduction

*Caladium bicolor*, a perennial foliage plant of the genus *Caladium* (Araceae), is native to tropical South America [1]. With its vibrant leaf coloration, distinctive morphology, and exceptional ornamental value, *C. bicolor* has experienced steadily increasing demand in domestic and international markets, establishing itself as a commercially important ornamental species [2,3]. However, its tropical origin renders it highly sensitive to environmental conditions, making it particularly vulnerable to abiotic stresses such as drought and cold during cultivation. These stresses frequently cause growth inhibition, leaf wilting, and plant death [4], severely compromising its ornamental quality and limiting commercial-scale production. Therefore, elucidating the molecular mechanisms underlying the abiotic stress responses of *C. bicolor* and identifying key stress-tolerance genes are essential for breeding stress-tolerant cultivars and ensuring sustainable commercial cultivation.

Transcription factors serve as core regulatory components in plant stress response networks, modulating downstream gene expression through recognition of specific *cis*-acting elements to facilitate environmental adaptation [5,6]. Among transcription factor families, the basic helix–loop–helix (bHLH) family represents one of the largest and most functionally diverse groups in plants. bHLH proteins typically contain a highly conserved bHLH domain comprising a DNA-binding region rich in basic amino acids and two α-helices connected by a loop [7]. The basic region recognizes and binds *cis*-acting elements such as E-box (CANNTG) or G-box (CACGTG), while the helical structures mediate homo- or heterodimerization, thereby regulating downstream gene expression [8,9]. Based on phylogenetic relationships and structural features, plant bHLH transcription factors are classified into multiple subfamilies; for instance, the *Arabidopsis thaliana* bHLH family comprises 31 subfamilies [10]. Members of these subfamilies play crucial roles in diverse biological processes, including growth and development, hormone signal transduction, secondary metabolism, and abiotic stress responses [11,12].

In model plants and major crops such as *Arabidopsis*, rice, and maize, several bHLH members have been shown to significantly enhance tolerance to drought, cold, and salt stress by regulating antioxidant systems, osmolyte biosynthesis, and stress-related gene expression [13,14]. Furthermore, bHLH transcription factors function as key nodes integrating multiple hormone signaling pathways, including abscisic acid (ABA), jasmonic acid (JA), and salicylic acid (SA), to coordinately regulate stress responses [15,16,17]. For example, *Arabidopsis AtbHLH122* enhances drought tolerance through ABA signaling modulation [18], while rice *OsbHLH148* participates in JA-mediated drought adaptation [19,20]. Transgenic poplar overexpressing *PdbbHLH1* exhibited improved drought tolerance via enhanced antioxidant enzyme activity and upregulation of drought-responsive genes [21]. Hairy root transformation experiments in grapevine demonstrated that *VvbHLH036* overexpression increased cold tolerance [22]. In pepper, *CabHLH035* promotes cold tolerance and maintains reactive oxygen species (ROS) homeostasis [23]; furthermore, its transient overexpression enhanced salt tolerance, whereas its silencing reduced it [24]. Despite extensive functional characterization in major crops and model plants, research on ornamental species, particularly Araceae members, remains limited.

In this study, we successfully cloned a bHLH transcription factor gene from *C. bicolor*, designated *CbbHLH35*, and systematically characterized its structural features and biological functions. Initially, multiple sequence alignments and phylogenetic analyses were performed to elucidate the evolutionary position and conserved domains of the CbbHLH35 protein. Subcellular localization assays subsequently revealed its intracellular distribution. Furthermore, quantitative real-time PCR (qRT-PCR) was employed to comprehensively profile the spatiotemporal expression patterns of *CbbHLH35* across distinct tissues, as well as its responses to exogenous hormones and diverse abiotic stresses. Finally, we evaluated the physiological performance of *CbbHLH35*-overexpressing *C. bicolor* lines under drought and cold stress conditions. Collectively, this study not only unveils the role of *CbbHLH35* in stress adaptation, thereby enriching the bHLH functional database in *C. bicolor*, but also provides essential genetic resources and theoretical foundations for the molecular breeding of stress-tolerant cultivars.

## 2. Results

### 2.1. Sequence Alignment and Phylogenetic Analysis of CbbHLH35

**Figure 1:**
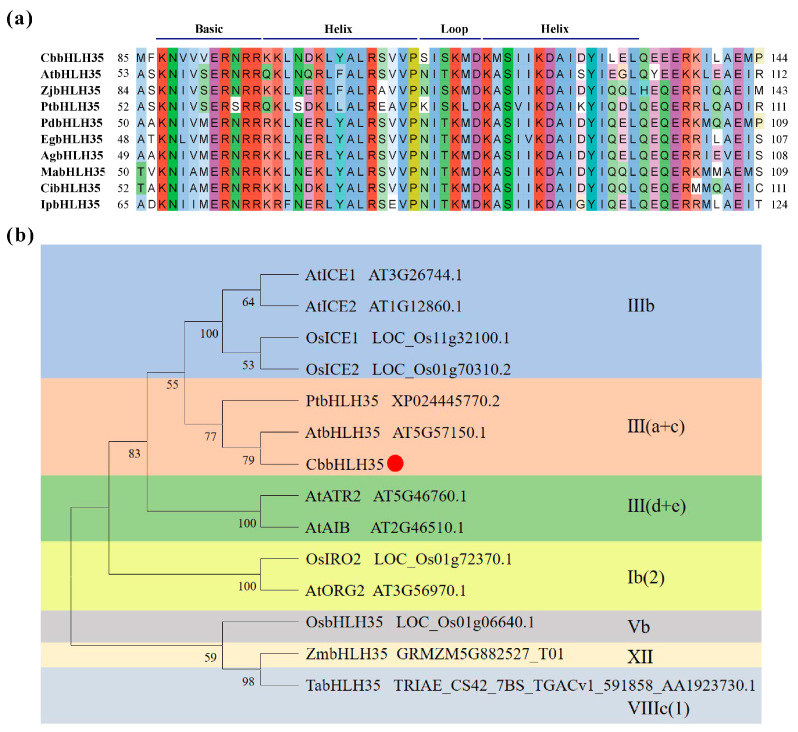
Multiple sequence alignment and phylogenetic analysis of CbbHLH35 from *C. bicolor*. (**a**) Multiple sequence alignment of CbbHLH35 with bHLH35 homologous proteins from different plant species. Conserved amino acid residues within the canonical basic helix–loop–helix (bHLH) domain are highlighted in the alignment. The numbers on the left and right indicate the amino acid positions of each sequence. (**b**) Phylogenetic tree constructed based on bHLH protein sequences from representative plant species. CbbHLH35 is indicated by a red dot in the tree. Species abbreviations are as follows: Cb, *Caladium bicolor*; At, *Arabidopsis thaliana*; Zj, *Ziziphus jujuba;* Pt, *Populus tomentosa*; Pd, *Phoenix dactylifera*; Eg, *Elaeis guineensis*; Ag, *Acorus gramineus*; Ma, *Musa acuminata*; Ci, *Canna indica*; Ip, *Iris pallida*; Os, *Oryza sativa*; Zm, *Zea mays*; Ta, *Triticum aestivum*. The phylogenetic tree was constructed using the Maximum Likelihood method with 1000 bootstrap replicates in MEGA (version 11.0).

To characterize the structural features of CbbHLH35, multiple sequence alignment was performed using bHLH35 homologous proteins from *Arabidopsis thaliana*, *Ziziphus jujuba*, *Populus tomentosa*, *Phoenix dactylifera*, *Elaeis guineensis*, *Acorus gramineus*, *Musa acuminata*, *Canna indica*, and *Iris pallida* (Figure 1a). The results showed that CbbHLH35 contains a typical and highly conserved basic helix–loop–helix (bHLH) domain. This domain consists of a basic region enriched in basic amino acids responsible for DNA binding, followed by two α-helices separated by a loop region. Key amino acid residues in the basic region, particularly arginine (R) and lysine (K), were highly conserved among the analyzed species, indicating their critical role in DNA-binding activity. Furthermore, the two helix regions exhibited high sequence conservation between monocotyledonous and dicotyledonous plants, suggesting that the structural framework of this transcription factor has been evolutionarily conserved.

To further elucidate the evolutionary relationship of CbbHLH35, bHLH35 protein sequences from representative monocot and dicot species, including *Arabidopsis thaliana*, *Oryza sativa*, *Populus tomentosa*, *Zea mays*, and *Triticum aestivum*, were selected. In addition, several stress-responsive bHLH proteins from *Arabidopsis* and rice were included for phylogenetic analysis [25], and a phylogenetic tree was constructed (Figure 1b). The phylogenetic analysis revealed that CbbHLH35 clustered within the III(a + c) subfamily of the bHLH transcription factor family. It formed a strongly supported clade with PtbHLH35 and AtbHLH35, indicating a close evolutionary relationship within this subgroup. As members of the III(a + c) subfamily are known to participate in the regulation of plant development and abiotic stress responses, the phylogenetic placement of CbbHLH35 implies that it may perform a comparable regulatory function in *C. bicolor.*

### 2.2. CbbHLH35 Is Specifically Localized in the Nucleus

To determine the subcellular localization of *CbbHLH35* in plant cells, a 35S::*CbbHLH35*::GFP fusion construct was generated and transiently expressed in *Nicotiana benthamiana* leaf epidermal cells via *Agrobacterium*-mediated transformation. The 35S::GFP empty vector served as a control.

**Figure 2:**
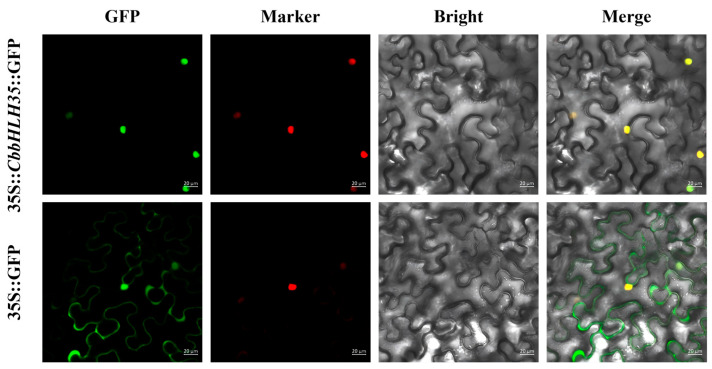
Subcellular localization of *CbbHLH35*. The 35S::*CbbHLH35*::GFP fusion construct was transiently expressed in *N. benthamiana* leaf epidermal cells. The 35S::GFP vector was used as a control. GFP indicates green fluorescence signals, while VirD2NLS-mCherry represents the red fluorescent nuclear marker. Bright indicates bright-field images, and Merge represents the merged fluorescence images. Scale bar = 20 μm.

Confocal laser scanning microscopy revealed that in the control group (35S::GFP), the green fluorescence signal was distributed throughout the entire cell, including both the cytoplasm and nucleus, indicating a non-specific intracellular distribution of free GFP (Figure 2). In contrast, in the experimental group (35S::*CbbHLH35*::GFP), the green fluorescence signal was predominantly confined to the nucleus and showed strong overlap with the red fluorescent nuclear marker (VirD2NLS-mCherry). The merged images further confirmed the co-localization of the GFP signal with the nuclear marker. Therefore, the specific nuclear localization of *CbbHLH35* further supports the hypothesis that it functions as a transcriptional regulator involved in gene expression control.

### 2.3. Tissue-Specific Expression Patterns of CbbHLH35

**Figure 3:**
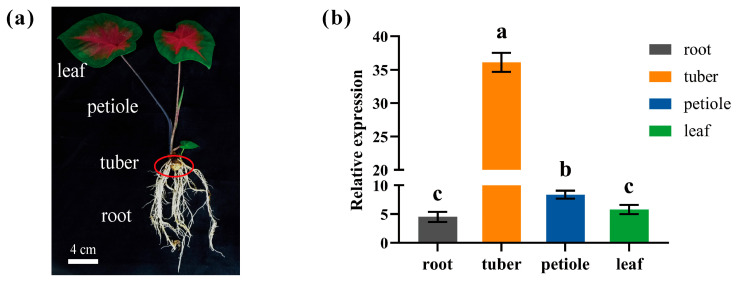
Phenotypic characteristics of different tissues and expression analysis of *CbbHLH35* in *C. bicolor*. (**a**) Phenotypes of leaf, petiole, tuber, and root tissues in *C. bicolor* ‘Hongtao K’. Scale bar = 4 cm. (**b**) Relative expression levels of *CbbHLH35* in different tissues. Each group included three biological and three technical replicates, with data presented as the mean ± standard deviation (SD). Different lowercase letters indicate significant differences (*p* < 0.05).

To characterize the expression pattern of *CbbHLH35* in different tissues of *C. bicolor*, root, tuber, petiole, and leaf tissues were collected for qRT-PCR analysis. The plant phenotype of *C. bicolor* ‘Hongtao K’ and its tissue phenotypes are shown in Figure 3. Expression analysis revealed that *CbbHLH35* was expressed in all examined tissues, although its transcript abundance varied significantly among them (Figure 3b). Notably, *CbbHLH35* exhibited the highest expression level in the tuber, which was significantly higher than that in the other tissues (*p* < 0.05). The expression level in the petiole was moderate, whereas relatively low transcript levels were detected in both root and leaf tissues, with no significant difference observed between these two tissues. Collectively, these results indicate that *CbbHLH35* is highly expressed in tuber tissue, suggesting that it may play an important regulatory role in tuber development or associated physiological processes.

### 2.4. Expression Patterns of CbbHLH35 Under Phytohormone Treatments and Abiotic Stresses

**Figure 4:**
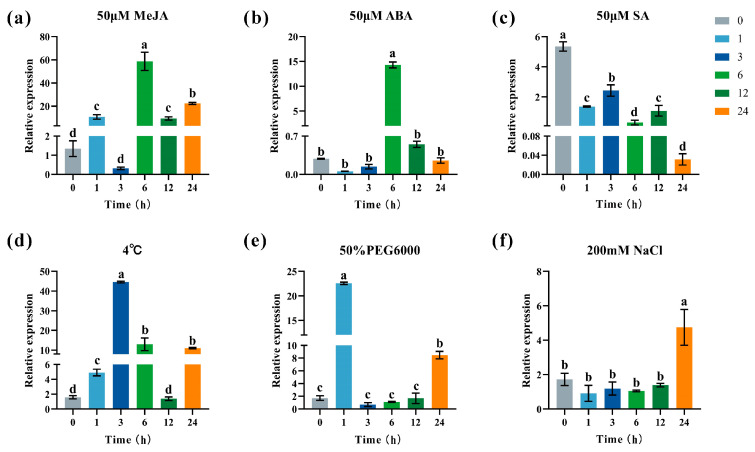
Expression analysis of *CbbHLH35* under different phytohormone treatments and abiotic stress conditions. (**a**) 50 μM MeJA treatment; (**b**) 50 μM ABA treatment; (**c**) 50 μM SA treatment; (**d**) 4 °C cold treatment; (**e**) 50% (*w*/*v*) PEG6000-induced drought treatment; (**f**) 200 mM NaCl treatment. Different lowercase letters indicate significant differences among time points within the same treatment (*p* < 0.05).

To investigate the responsiveness of *CbbHLH35* to various phytohormones and abiotic stresses, *C. bicolor* plants were treated with 50 μM MeJA, 50 μM ABA, 50 μM SA, cold stress (4 °C), 50% (*w*/*v*) PEG6000, and 200 mM NaCl. The expression levels were examined at different time points (Figure 4).

The results showed that *CbbHLH35* responded to multiple hormone treatments and stress conditions to varying degrees, with distinctly different induction patterns. Under MeJA treatment, *CbbHLH35* expression was significantly upregulated as early as 1 h and reached a peak at 6 h (43.51-fold relative to 0 h). Subsequently, the expression level declined, but it remained significantly higher than the 0 h level at 24 h, indicating that this gene is highly sensitive to MeJA signaling (Figure 4a). ABA treatment also significantly induced *CbbHLH35* expression, which reached its highest level at 6 h (50.34-fold relative to 0 h), followed by a gradual decrease (Figure 4b). Under SA treatment, the expression levels of *CbbHLH35* were significantly lower than those at 0 h at all examined time points, showing no obvious induction effect (Figure 4c). Cold treatment (4 °C) significantly promoted *CbbHLH35* expression, which peaked at 3 h (28.20-fold relative to 0 h), recovered to the control level at 12 h, and significantly increased again at 24 h (Figure 4d). Under PEG6000-simulated drought treatment, *CbbHLH35* was rapidly induced at 1 h, reaching 13.12-fold of the 0 h level (Figure 4e). Subsequently, the expression level decreased significantly, showing no significant difference from the control between 3 and 12 h, but it was significantly upregulated again at 24 h. In contrast, under NaCl treatment, *CbbHLH35* showed no significant changes within 1–12 h but was significantly upregulated at 24 h (Figure 4f). Overall, *CbbHLH35* exhibited significant and rapid induction responses to MeJA, ABA, cold, and drought treatments, suggesting that it may be involved in the regulatory processes of multiple hormone signaling pathways and abiotic stress response networks.

### 2.5. Overexpression of CbbHLH35 Enhances Drought and Cold Tolerance in C. bicolor

**Figure 5:**
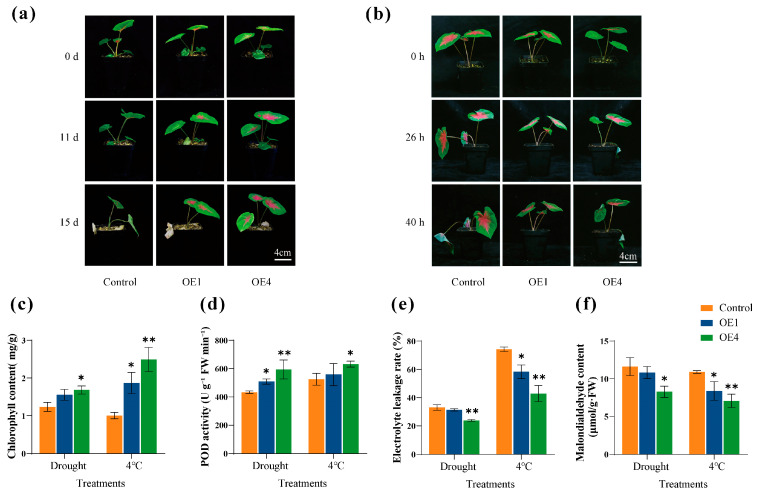
Phenotypic and physiological analyses of *CbbHLH35* transgenic *C. bicolor* under natural drought and low-temperature stress. Phenotypic comparison between non-transgenic lines (Control) and overexpression lines (OE1 and OE4) after natural drought treatment for 25 days (**a**) and low-temperature treatment at 4 °C for 40 h (**b**). Scale bar = 4 cm. (**c**) Chlorophyll content; (**d**) peroxidase (POD) activity; (**e**) electrolyte leakage rate; (**f**) malondialdehyde (MDA) content. Statistical analysis was performed using multiple *t*-tests, with the control plants as the reference group. Data are presented as the mean ± standard deviation (SD). (* *p* < 0.05, ** *p* < 0.01).

To further verify the role of *CbbHLH35* in drought and low-temperature stress responses, the control group (Control) and overexpression plants (OE1 and OE4) were subjected to 25 days of natural drought treatment and 40 h of low-temperature treatment at 4 °C, respectively. The phenotypes and related physiological indices were subsequently analyzed (Figure 5).

The results showed that after 25 days of natural drought treatment, the control group exhibited severe wilting, leaf drooping, and drying symptoms, whereas OE1 and OE4 displayed significantly less damage and maintained better overall growth status than the control (Figure 5a). After 40 h of low-temperature treatment, the control group showed obvious water loss and wilting, while the overexpression lines maintained relatively normal leaf morphology (Figure 5b). Under both drought and low-temperature stress, the chlorophyll content of the OE lines was higher than that of the control, with OE4 showing a significantly higher chlorophyll content (Figure 5c). Under drought conditions, the peroxidase (POD) activity in the OE lines was significantly higher than that in the control group, and this increase was particularly pronounced in OE4 under low-temperature stress (Figure 5d). Meanwhile, the electrolyte leakage rate and malondialdehyde (MDA) content of the OE lines were lower than those of the control under both drought and low-temperature treatments (Figure 5e,f). Specifically, under drought stress, OE4 showed a significant decrease compared with the control, while under low-temperature stress, both OE1 and OE4 exhibited a significant decrease. These results indicate that the OE lines possessed higher membrane stability and lower levels of lipid peroxidation. Taken together, compared with the control plants, OE1 and OE4 exhibited higher chlorophyll content and POD activity, as well as lower electrolyte leakage and MDA content under drought and low-temperature stresses. This effectively alleviated stress-induced cellular damage and enhanced overall plant stress tolerance, with OE4 displaying a relatively stronger tolerance to both stresses.

## 3. Discussion

bHLH transcription factors play important roles in the complex regulatory networks that govern plant growth, development, and adaptation to environmental stress. Although members of the bHLH family have been extensively studied in *Arabidopsis* [10], rice [14], and maize [26,27], their functions in foliage ornamental plants remain largely unknown. In *C. bicolor*, *CbbHLH35* exhibited a broad yet tissue-specific expression pattern across roots, tubers, petioles, and leaves, with the highest transcript abundance detected in tubers. Similar tissue-preferential expression patterns have also been reported in other species. For example, *PabHLH35* is abundantly expressed in the shoot apex, axillary buds, and young spikes of *Phalaenopsis Aphrodite* [28], whereas *OsbHLH035* is highly expressed in root tips, vascular tissues, and lateral roots of rice [29]. As a perennial species, *C. bicolor* relies on tubers as primary storage organs for nutrient accumulation and survival under adverse conditions. The preferential expression of *CbbHLH35* in tubers thus implicates its potential role in maintaining physiological homeostasis and mediating adaptation to environmental fluctuations.

To further investigate the role of *CbbHLH35* in hormone responses and other abiotic stresses, *C. bicolor* plants were subjected to a range of hormone and stress treatments. qRT-PCR analysis showed that *CbbHLH35* responded to MeJA, ABA, SA, low-temperature, PEG-induced drought, and salt treatment, with particularly strong induction under MeJA, ABA, drought, and low-temperature conditions. ABA is widely recognized as a central hormone in drought stress and rewatering responses, as demonstrated in potato [30], maize [31] and cotton [32]. In rice, *OsbHLH035* regulates seed germination and seedling recovery from salt stress through both ABA-dependent and ABA-independent pathways [29]. The ABA-dependent pathway generally activates downstream transcription factors via the core PYR/PYL–PP2C–SnRK2 signaling module, while ABA-independent pathways may involve MAPK cascades or other kinase-mediated phosphorylation processes, collectively contributing to a complex regulatory network governing plant stress responses [33]. MeJA is also closely associated with low-temperature responses [34], and in tomato it positively regulates chilling tolerance by promoting ABA biosynthesis [35]. In this context, the pronounced induction of *CbbHLH35* by ABA and MeJA implies that it may function at the nexus of hormonal and stress signaling networks, thereby coordinating adaptation to drought and cold stress. Further studies are needed to clarify the coordinated regulation between *CbbHLH35* and hormone signaling pathways during abiotic stress responses. Protein–protein interaction assays (e.g., Y2H, BiFC, and Co-IP) could be used to identify potential interacting partners and determine whether *CbbHLH35* regulates downstream gene expression by forming complexes with key regulators in ABA or MeJA signaling (e.g., ABFs or JAZ proteins).

Functional analyses further supported a positive role of *CbbHLH35* in stress tolerance. Under both drought and low-temperature stress, the overexpression lines OE1 and OE4 exhibited markedly better growth performance than the control plants. After 25 d of natural drought treatment, control plants displayed severe wilting, leaf drooping, and desiccation, whereas the overexpression lines maintained a relatively normal growth phenotype and showed less damage [4]. Similarly, after 40 h at 4 °C, control plants showed obvious water loss and wilting, whereas the overexpression lines retained better leaf morphology [36]. These phenotypic differences indicate that overexpression of *CbbHLH35* enhances drought and low-temperature tolerance in *C. bicolor*. Physiological data further suggest that *CbbHLH35* enhances stress tolerance through the coordinated regulation of multiple protective processes. Under drought and low-temperature stress, the overexpression lines maintained higher chlorophyll contents than the control, with OE4 showing a significant increase, indicating that overexpression of *CbbHLH35* may alleviate stress-induced chlorophyll degradation and help maintain photosynthetic stability [4]. Meanwhile, the OE lines exhibited higher POD activity under both stress conditions, with the most pronounced increase observed in OE4 under low temperature, suggesting that the antioxidant defense system was more effectively activated and that ROS-scavenging capacity was enhanced [37,38]. In pepper, *CabHLH035* functions as an upstream transcription factor that directly activates the downstream target gene *CaAPX*, thereby promoting the production of ascorbate peroxidase (APX) and effectively participating in the scavenging of reactive oxygen species (ROS). In addition, *CabHLH035* activates the key regulator *CaCBF1A* in the cold signaling pathway. These two pathways act synergistically to reduce oxidative damage and membrane disruption induced by cold stress, ultimately enhancing plant cold tolerance [23]. Based on the physiological and gene expression analysis results of this study, it is speculated that *CbbHLH35* may participate in the abiotic stress response process by regulating antioxidant-related genes (such as POD) and hormone signaling pathways. Furthermore, electrolyte leakage and MDA content were both lower in the overexpression lines than in the control, indicating reduced membrane damage and weaker lipid peroxidation under stress conditions. Reduced electrolyte leakage reflects better maintenance of membrane stability, whereas decreased MDA accumulation further supports attenuation of oxidative damage to cellular membranes [36]. Together, these results suggest that *CbbHLH35* may improve drought and low-temperature tolerance in *C. bicolor* by maintaining photosynthetic function, strengthening antioxidant defense, and preserving membrane integrity.

Taken together, our results establish *CbbHLH35* as a crucial positive regulator of abiotic stress responses in *C. bicolor*, providing a foundation for dissecting its role in integrating hormonal signaling and stress-responsive regulatory networks and highlighting its potential as a promising target for molecular breeding of stress-tolerant ornamental plants.

## 4. Materials and Methods

### 4.1. Multiple Sequence Alignment and Phylogenetic Analysis

The bHLH35 protein sequences of *C. bicolor*, *Arabidopsis thaliana*, *Ziziphus jujuba*, *Populus tomentosa*, *Phoenix dactylifera*, *Elaeis guineensis*, *Acorus gramineus*, *Musa acuminata*, *Canna indica*, and *Iris pallida* were retrieved from the National Center for Biotechnology Information (NCBI) website (https://www.ncbi.nlm.nih.gov/, accessed on 15 January 2022). Multiple sequence alignments were performed using the MUSCLE algorithm implemented in MEGA (version 11.0) with default parameters. The alignments were subsequently visualized and manually inspected using Jalview (version 2.11.2.6) to evaluate sequence conservation and identify conserved domains.

To investigate the evolutionary relationship of bHLH35 proteins, a phylogenetic tree was constructed using MEGA software (version 11.0). The bHLH35 protein of *C. bicolor* was analyzed together with homologous sequences from *Arabidopsis thaliana*, *Oryza sativa*, *Populus tomentosa*, *Zea mays*, and *Triticum aestivum*, as well as several previously reported stress-responsive bHLH proteins [25]. The phylogenetic tree was generated using the Maximum Likelihood (ML) method with 1000 bootstrap replicates to assess node reliability. The best-fit substitution model was selected based on the lowest Bayesian Information Criterion (BIC) score. The final tree was annotated and visualized using the Interactive Tree Of Life (iTOL) online tool (http://itol.embl.de/index.shtml, accessed on 20 September 2023).

### 4.2. Cloning of CbbHLH35

Total RNA was extracted from *C. bicolor* leaves using the EASYspin Plant RNA Rapid Extraction Kit (Boer, Beijing, China). First-strand cDNA was synthesized using the PrimeScript RT Reagent Kit with gDNA Eraser (TaKaRa, Dalian, China) according to the manufacturer’s instructions. Based on the coding sequence (CDS) of *CbbHLH35* obtained from our previous transcriptome data [39], *CbbHLH35*-specific primers (*CbbHLH35*-F/R, Table S1) were designed using Primer Premier software (version 5.0). PCR amplification was performed using TransStart^®^ Top Taq DNA Polymerase (TransGen, Beijing, China) under the following conditions: an initial denaturation at 94 °C for 5 min; followed by 30 cycles of 94 °C for 30 s, 57 °C for 30 s, and 72 °C for 100 s; and a final extension at 72 °C for 10 min. The purified amplicons were cloned into the pMD19-T vector (Takara, Shiga, Japan) and verified by sequencing.

### 4.3. Subcellular Localization Analysis of CbbHLH35

To determine the subcellular localization of *CbbHLH35*, the CDS of *CbbHLH35* without the stop codon was amplified and inserted into the pCAMBIA1300-GFP vector to generate a *CbbHLH35*–GFP fusion driven by the CaMV 35S promoter. The resulting 35S::*CbbHLH35*–GFP construct and the pCAMBIA1300-GFP empty vector were separately introduced into *Agrobacterium tumefaciens* strain GV3101 (Weidibio, Shanghai, China). *Agrobacterium* suspensions harboring the constructs were infiltrated into *Nicotiana benthamiana* leaves for transient expression. The nuclear marker VirD2NLS-mCherry was co-infiltrated to label the nucleus. Following infiltration, plants were kept in the dark for 24–48 h. The infiltrated leaf epidermal cells were then examined for fluorescence signals using a laser scanning confocal microscope.

### 4.4. Expression Analysis of CbbHLH35

Three-month-old tissue-cultured plants of *C. bicolor* cultivar ‘Hongtao K’ were used to profile the expression patterns of *CbbHLH35*. The plants were grown under controlled conditions at 25 °C, 70% relative humidity, a light intensity of 60 µmol·m^−2^·s^−1^ (cool white fluorescent lamps), and a 16 h light/8 h dark photoperiod [39].

For tissue-specific expression analysis, roots, stems, tubers, leaves, and petioles were collected from plants three months after transplantation. All samples were immediately flash-frozen in liquid nitrogen and stored at −80 °C. Three biological replicates were performed for each sample, and each biological replicate included three technical replicates.

To investigate the expression responses of *CbbHLH35* under hormone treatments and abiotic stresses, control plants (*C. bicolor* ‘Hongtao K’) and transgenic lines (OE1 and OE4) with uniform growth were selected for various treatments. For hormone treatments, leaves were sprayed with 50 μM methyl jasmonate (MeJA), 50 μM salicylic acid (SA), or 50 μM abscisic acid (ABA) solutions until runoff. Drought stress was simulated by irrigating plants with 50% (*m*/*v*) PEG6000 solution, while salt stress was induced using 200 mM NaCl solution. Leaf samples were collected at 0, 1, 3, 6, 12, and 24 h post-treatment, immediately frozen in liquid nitrogen, and stored at −80 °C.

Gene-specific primers for qRT-PCR were designed using Primer Premier 5.0 (Table S1). Three biological replicates and three technical replicates were included for each treatment at each sampling time point. qRT-PCR was performed on a Bio-Rad CFX96 system using SsoFast™ EvaGreen^®^ Supermix (Bio-Rad, Hercules, CA, USA) according to the manufacturer’s instructions. The PCR program consisted of an initial denaturation at 95 °C for 3 min, followed by 40 cycles of 95 °C for 5 s, 60 °C for 5 s, and 72 °C for 5 s, with a final melting curve analysis from 65 °C to 95 °C. The q*CbUBC* gene [40] was used as the internal reference for normalization, and relative gene expression levels were calculated using the 2^−ΔΔCt^ method [41]. Statistical analysis was performed using IBM SPSS Statistics software (version 27.0.1.1; SPSS, Chicago, IL, USA) with one-way analysis of variance (ANOVA), and bar charts were generated using GraphPad Prism software (version 9.5.0; GraphPad Software, San Diego, CA, USA).

### 4.5. Generation of Transgenic C. bicolor Plants

The CDS of *CbbHLH35* was cloned into the pCAMBIA1300 plant overexpression vector containing the CaMV 35S promoter using *Kpn* I and *Sal* I restriction sites. Specific primers (*1300-CbbHLH35*-F/R) were designed based on homologous recombination principles (Table S1), and the 35S::*CbbHLH35* expression vector was successfully constructed. The recombinant plasmid was transformed into *A. tumefaciens* strain GV3101 (Weidibio, Shanghai, China) via electroporation.

The recombinant GV3101 strain carrying the 35S::*CbbHLH35* construct was cultured in YEB liquid medium until the OD600 reached 0.6–0.8. The bacterial cells were then collected by centrifugation and resuspended in MS infection medium, and the suspension was adjusted to OD600 = 0.3 for infection. Agrobacterium-mediated transformation was subsequently performed in *C. bicolor* leaves using the leaf-disc infection method [39,42].

The infected leaf discs were cultured on MS medium supplemented with 4.0 mg/L 6-benzylaminopurine (6-BA), 0.2 mg/L 1-naphthaleneacetic acid (NAA), and 50 mg/L hygromycin for transgenic selection. Genomic DNA was extracted from the leaves of putative transgenic plants and control plants (*C. bicolor* ‘Hongtao K’) for molecular identification. PCR amplification was performed using *CbbHLH35*-specific primers (*CbbHLH35* F/R) (Table S1), and the PCR products were analyzed by 1% agarose gel electrophoresis, following the PCR program described above. The 35S::*CbbHLH35* plasmid was used as the positive control, while control plants (*C. bicolor* ‘Hongtao K’) served as the negative control. The electrophoresis results confirmed the successful integration of the *CbbHLH35* gene into the genome of the transgenic *C. bicolor* plants.

### 4.6. Phenotypic Observation and Physiological Measurements of Transgenic Plants

To evaluate the impact of *CbbHLH35* overexpression on stress tolerance in *C. bicolor*, two independent T2 transgenic lines exhibiting high expression levels (OE1 and OE4) (Figure S1) were selected for further analysis, and control plants (*C. bicolor* ‘Hongtao K’) were selected for phenotypic analysis. Tissue-cultured plantlets were transplanted and grown for two months, after which plants with uniform growth were subjected to stress treatments.

Drought stress was induced by withholding irrigation after thoroughly watering the plants one day prior to treatment. Plants were maintained under controlled conditions at 25 °C with 70% relative humidity, a light intensity of 60 µmol·m^−2^·s^−1^, and a 16 h light/8 h dark photoperiod [39]. Cold stress was applied in a climate chamber at 4 °C under otherwise identical conditions.

Leaf samples were collected when visible stress symptoms appeared and used for physiological measurements. All physiological parameters were determined using fresh leaf tissue, with three biological and three technical replicates. Chlorophyll content was measured from 0.1 g of fresh tissue using the ethanol–acetone extraction method [43,44], while peroxidase (POD) activity was assayed from 0.3 g of fresh tissue using the guaiacol method [45]. For electrolyte leakage, 0.1 g of fresh leaf tissue was cut into small pieces and incubated in ddH_2_O at room temperature for 3 h. Initial conductivity (R1) was measured, followed by boiling for 30 min, cooling to room temperature, and re-measurement of final conductivity (R2). Electrolyte leakage was calculated as RC (%) = (R1/R2) × 100% [46]. Malondialdehyde (MDA) content was determined following a previously described method [47]. Leaf samples (0.5 g) were homogenized in 5 mL of 5% (*m*/*v*) trichloroacetic acid (TCA) and centrifuged at 4000 rpm for 10 min. The supernatant (2 mL) was mixed with an equal volume of 0.67% (*m*/*v*) thiobarbituric acid (TBA) and heated at 100 °C for 30 min, followed by rapid cooling and centrifugation. Absorbance was measured at 532, 450, and 600 nm. MDA concentration (C_MDA_) was calculated as 6.45 *(OD_532_ − OD_600_) − 0.559 * OD_450_. MDA content was expressed as μmol g^−1^ fresh weight (FW) and calculated as C_MDA_ *N *W^−1^, where N represents the volume of the TCA extract and W represents the fresh weight.

## 5. Conclusions

This study systematically analyzed the molecular characteristics, expression patterns, and biological functions of *CbbHLH35* in *Caladium bicolor*. CbbHLH35 contains a conserved bHLH domain and is localized in the nucleus. It is expressed in different tissues, with the highest expression detected in tubers, and is significantly induced by MeJA, ABA, drought, and low-temperature treatments. Overexpression of *CbbHLH35* enhanced the tolerance of *C. bicolor* to drought and cold stress. Taken together, these findings suggest that *CbbHLH35* positively regulates abiotic stress tolerance, thereby offering a potential target for genetic improvement and the molecular breeding of stress-tolerant *C. bicolor* cultivars. Future studies should dissect its regulatory network and downstream targets to unravel the molecular mechanisms driving *CbbHLH35*-mediated stress tolerance.

## Data Availability

Data are contained within the article and Supplementary Materials.

## Supplementary Materials

The following supporting information can be downloaded at https://www.mdpi.com/article/10.3390/plants15071120/s1, Table S1: List of primers; Figure S1: Relative expression levels of *CbbHLH35* in different *Caladium bicolor* lines.
