# Supplementary material for: Functional Analysis of CbbHLH35 Reveals Its Role in Drought and Cold Stress Tolerance in Caladium bicolor
Source: Plants (Basel). 2026 Apr 6;15(7):1120. doi: 10.3390/plants15071120 (PMC13075155; doi:10.3390/plants15071120)
Supplement: Supplementary file 1 [file plants-15-01120-s001.zip › Table S1.pdf]

Table S1. List of primers

Primer name	Primer sequences (5′→3′)
CbbHLH35-F	GAGCATGGATCTACTGGAAGC
CbbHLH35-R	ACCTCGGTCTAATTAAGCACG
1300-CbbHLH35-F	ggacgagctcggtaccATGGATCTACTGGAAGCTGGGG
1300-CbbHLH35-R	cccttgctcaccatgtcgacATTAAGCACGTGTACACTCGTG
qCbUBC-F	CCGAAGGTCCGCTTTCTTACAA
qCbUBC-R	CTCCTTTGCTGTTTCTACGGCT
qCbbHLH35-F	CCTTTCGGCCAACATCGC
qCbbHLH35-R	GGCACGCATCAAGTTCAGC
