# Supplementary figures and images for: Functional Analysis of CbbHLH35 Reveals Its Role in Drought and Cold Stress Tolerance in Caladium bicolor
Source: Plants (Basel). 2026 Apr 6;15(7):1120. doi: 10.3390/plants15071120 (PMC13075155; doi:10.3390/plants15071120)

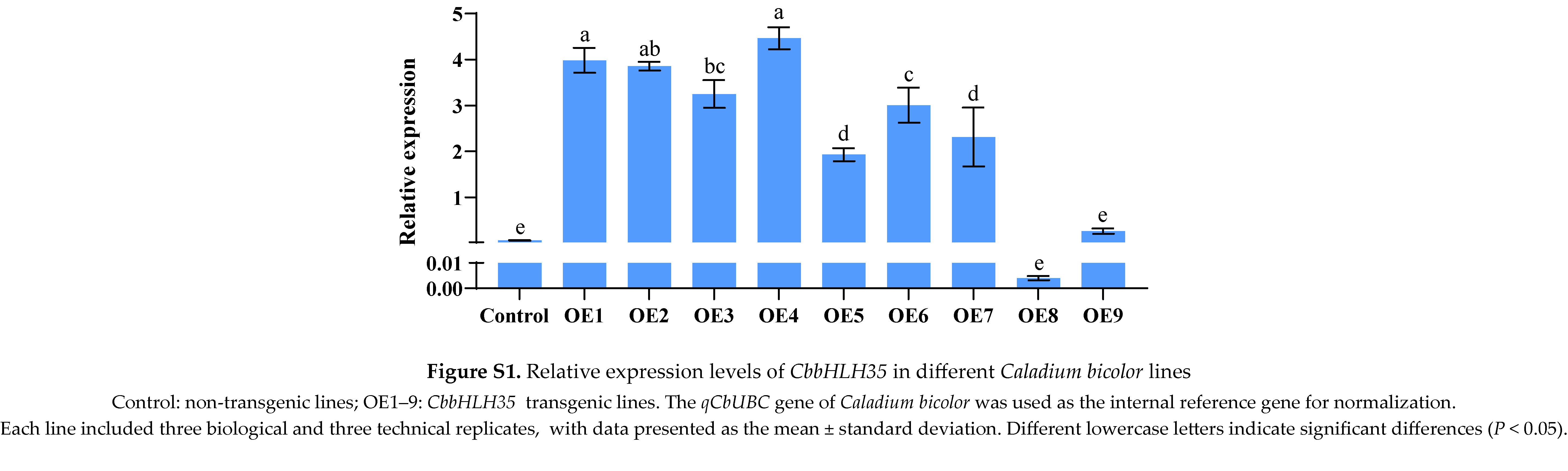

Supplement: Supplementary file 1 [file plants-15-01120-s001.zip › Figure S1.jpg]
